# Simultaneous Casualty Admissions—Do they Affect Treatment in the Receiving Trauma Center?

**DOI:** 10.1007/s00268-021-06074-8

**Published:** 2021-03-29

**Authors:** Michel Paul Johan Teuben, Carsten Mand, Laura Moosdorf, Kai Sprengel, Alba Shehu, Roman Pfeifer, Steffen Ruchholtz, Rolf Lefering, Hans-Christoph Pape, Kai Oliver Jensen

**Affiliations:** 1 Department of Trauma, University Hospital Zurich, Raemistrasse 100, 8090 Zurich, Switzerland; 2 Department of Surgery, Thurgau Cantonal Hospital, Frauenfeld, Switzerland; 3 Department of Trauma-,Hand-, and Reconstructive Surgery, University Clinic Giessen and Marburg, Marburg, Germany; 4 Institute for Research in Operative Medicine (IFOM), University Witten/Herdecke, Cologne, Germany

## Abstract

**Background:**

Simultaneous trauma admissions expose medical professionals to increased workload. The impact of simultaneous trauma admissions on hospital allocation, therapy, and outcome is currently unclear. We hypothesized that multiple admission-scenarios impact the diagnostic pathway and outcome.

**Methods:**

The TraumaRegister DGU® was utilized. Patients admitted between 2002–2015 with an ISS ≥ 9, treated with ATLS®- algorithms were included. Group ´IND´ included individual admissions, two individuals that were admitted within 60 min of each other were selected for group ´MULT´. Patients admitted within 10 min were considered as simultaneous (´SIM´) admissions. We compared patient and trauma characteristics, treatment, and outcomes between both groups.

**Results:**

132,382 admissions were included, and 4,462/3.4% MULTiple admissions were found. The SIM-group contained 1,686/1.3% patients. The overall median injury severity score was 17 and a mean age of 48 years was found. MULT patients were more frequently admitted to level-one trauma centers (68%) than individual trauma admissions were (58%, p < 0.001). Mean time to CT-scanning (24 vs. 26/28 min) was longer in MULT / SIM patients compared to individual admissions. No differences in utilization of damage control principles were seen. Moreover, mortality rates did not differ between the groups (13.1% in regular admissions and 11.4%/10,6% in MULT/SIM patients).

**Conclusion:**

This study demonstrates that simultaneous treatment of injured patients is rare. Individuals treated in parallel with other patients were more often admitted to level-one trauma centers compared with individual patients. Although diagnostics take longer, treatment principles and mortality are equal in individual admissions and simultaneously admitted patients. More studies are required to optimize health care under these conditions.

## Introduction

Simultaneous admission of trauma patients exposes institutions to an increased number and complexity of injuries and can lead to the overwhelming of institutions´ capacities. A situation is defined as a mass casualty incident (MCI) if the number of patients and complexity of cases exceeds the ability of an institution to provide adequate care. Preparedness for an MCI is essential to optimize outcome [1–4]. The global increase of mass events, mass shootings and terrorist attacks within the last decades has further increased the frequency of MCIs in civilian settings [5–10].

Treatment guidelines during MCIs are in line with advanced trauma life support® (ATLS) and damage control principles [11, 12]. However, the simultaneous admission of seriously-injured patients are not only characterized by increased medical demands, but also by logistical difficulties. Under normal conditions, the aim of healthcare providers is to provide optimal quality of care for admitted patients. In the case of multiple trauma cases, however, resources may be lacking to do so. So, during MCIs, the utilization of resources should be dosed, and only highly urgent diagnostics and interventions should be performed. This concept is known as minimal acceptable care [10, 13].

In the USA, up to 9% of trauma patients have been reported under MCI-scenarios. This does not appear to be so frequent in Europe, potentially due to a lower number of firearm incidents [13, 14]. As a readout for quality of care during MCIs in Europe, parallel treatment scenarios including seriously injured patients can be monitored. The outcome of patients admitted simultaneously with other trauma victims has not been studied in a multicenter setting before. Furthermore, the impact of multiple trauma cases on patient is unclear. We hypothesized that:

1. in multiple trauma admission scenarios (defined as > 1 seriously injured individual), patients are admitted more frequently to level-one trauma centers than under regular conditions.
2. The outcome in serious injured trauma victims admitted simultaneously with other trauma patients to a single institution is impaired compared with individual trauma admissions.

## Materials and methods

For this study, the TraumaRegister DGU® of the German Trauma Society (Deutsche Gesellschaft für Unfallchirurgie, DGU) was utilized. The aim of this multi-centre database is a pseudonymized and standardized documentation of severely injured patients.

Data are collected prospectively in four consecutive time phases from the site of the accident until discharge from hospital: (A) Pre-hospital phase, (B) Emergency room and initial surgery, (C) Intensive care unit and (D) Discharge. The documentation includes detailed information on demographics, injury pattern, comorbidities, pre- and in-hospital management, course on intensive care unit and outcome of each individual.

The infrastructure for documentation, data management and data analysis is provided by AUC—Academy for Trauma Surgery (AUC—Akademie der Unfallchirurgie GmbH), a company affiliated to the German Trauma Society. The scientific leadership is provided by the Committee on Emergency Medicine, Intensive Care and Trauma Management (Sektion NIS) of the German Trauma Society. The participating hospitals submit their data pseudonymized into a central database via a web-based application. Scientific data analysis is approved according to a peer review procedure laid down in the publication guideline of TraumaRegister DGU®.

The present study is in line with the publication guidelines of the TraumaRegister DGU® and registered as TR-DGU project ID 2018–024.

All trauma patients admitted to a German hospital between 2002 and 2015 with an ISS ≥ 9 and treated with ATLS®- algorithms were selected [15]. An ISS ≥ 9 represents serious injury, and this threshold was chosen based on recommendations from Palmer for trauma outcome studies focussing on more factors of interest than mortality only [16]. We excluded all transfer-in-patients (i.e., only primary admissions), early transfer-out patients (< 48 h; outcome unknown), and those individuals in which the exact time of hospital admission was unavailable. In addition, patients admitted simultaneously with more than 2 trauma patients were also excluded.

Injuries were classified according to the Abbreviated Injury Scale (AIS, version 2005, update 2008) and Injury Severity Scores (ISS) [15, 17, 18]. Neurological status was described by the Glasgow Come Scale [19]. Utilization of two established treatment concepts has been compared as well: early total care (ETC) and DCS (damage control surgery). Application of damage control surgery was defined as the need to execute damage control surgery for at least one of the encountered injuries. Indications for damage control surgery are described in the updated German polytrauma S3-guidelines. [20]. In accordance with current recommendations for analysis of large datasets on trauma and to optimize power of the study, outcome-adjusted comparisons using the prognostic RISC II score were performed [21].

Scenarios were divided based on the presence or absence of further admission of multiple trauma patients. The following study groups were composed:

Group IND: individual/regular trauma admissions.
Group MULT: patients admitted within 60 min after another trauma admission.
Sub-group SIM: patients admitted within 10 min after another trauma admission.

### Statistics

Continuous measurements were reported as mean with standard deviation (SD); and in case of skewed distribution of values, the median was reported in addition. ISS was documented as ISS (IQR). Categorical variables were reported as percentages with overall sample size. Due to the large group sizes, even minor differences would turn out to be statistically significant. Comparing p-values only is therefore not indicated [22]. Formal statistical testing was restricted to a few situations (chi-squared test for frequencies). Mortality rates were given with 95% confidence intervals (CI) in order to evaluate the difference to the prognosis (mean value derived from RISC II prognostic model). The RISC II is a prognostic score derived and validated with data from the TR-DGU [23]. Data were analyzed using SPSS Statistics Software (version 24, IBM Inc., Armonk, NY, USA).

## Results

A total of 200,804 patients were admitted to participating hospitals between 2002 and 2015. All patients with an ISS ≥ 9 treated in a German hospital were identified and included in the study if the date and time of admission were available (n = 147,965). Transferred patients (n = 15,402) and potential double entries (n = 181) were excluded, leaving 132,382 patients for analysis.

During the treatment of 2,200 seriously injured trauma patients (1.7% of 132,382), a second seriously injured trauma case has been admitted within one hour of admission. Another 62 additional patients were admitted within one hour. The total number of patients treated under ‘multiple patients’ conditions (MULT) was therefore 4,462 patients, representing 3.4% of all admissions. The subgroup of simultaneously admitted patients (admitted at the same time, or not more than 10 min later/SIM-group) consisted of 1686 cases (1.3%).

The mean age of the patients in the multiple admissions groups was 48.0 ± 22.3 years and 67.7% of patients were male. No relevant differences in patient-specific characteristics between groups were observed (Table 1).

**Table 1:** Patient and trauma characteristics

	Individual admissions (IND)	Multiple admissions (MULT)	Simultaneous admissions (SIM)
No. of patients	127,920	4,462 (3.4%)	1,686 (1.3%)
Age (years)	49.2 (22.1)	48.0 (22.3)	47.3 (22.4)
Sex (% males)	71.1%	67.7%	65.5%
*Injury type*			
Traffic accident (%)	55.8%	68.6%	73.8%
Blunt trauma (%)	95.6%	95.7%	94.8%
GCS ≤ 8	21.1%	17.6%	14.9%
Total ISS	17 (13–26)	18 (13–26)	17 (13–26)
*Relevant injuries* (AIS ≥ 3)			
Head	41.1%	37.0%	33.5%
Thorax	45.0%	49.0%	52.8%
Abdomen	12.3%	14.4%	15.4%
Extremities	30.4%	29.3%	30.1%

All data presented as mean (SD) or percentages. ISS is documented as median (IQR). Simultaneous admissions are a subgroup of the multiple admission group

The mean / median time difference between subsequent admissions was 22 / 18 min in the MULT-group. The MULT patients were more frequently involved in traffic accidents than regular admitted patients (68.6% vs. 55.8%). The individuals in the SIM-group were even more frequently involved in traffic accidents (73.8%).

The median (IQR) ISS of the whole cohort was 17 (13–26) points. No relevant differences in injury severity were seen between study groups (Table 1). Relevant head injuries were diagnosed less frequently in MULT patients (37% vs. 41%, p < 0.001), whereas thoracic injuries were seen more frequently (49% vs. 45%, p < 0.001). In subjects admitted simultaneously (group-SIM), relevant craniocerebral/cervical spine injuries were diagnosed in only 33.5% of cases and thoracic injuries occurred in 52.8% of the patients. Parallel to the lower rates of head injury, unconsciousness (GCS ≤ 8) was also less frequently observed in MULT cases (18%) and SIMultaneous (15%) admissions.

### Pre-hospital parameters and interventions

Individually admitted trauma patients were more frequently intubated out-of-hospital than MULT or SIM patients (33% vs. 29% vs. 25%). Pre-hospital sedation, chest-tube application and cardio-pulmonary resuscitation rates, however, did not differ between the groups.

Average transfer times from scene to hospital did not differ largely between the different conditions and varied between 63 and 65 min. Pre-hospital data are summarized in Table 2.

**Table 2:** Prehospital care

	Individual admissions	Multiple admissions	Simultaneous admissions
Intubation	32.5%	28.5%	24.5%
Sedation	71.0%	68.5%	69.5%
Chest-tube	4.3%	3.4%	3.1%
Cardio-pulmonary resuscitation (CPR)	3.3%	3.3%	2.5%
Volume therapy	87.0%	87.2%	86.4%
Total volume given (mL)	996 / 1000 (697)	976 / 1000 (681)	927 / 750 (635)
Total time from accident to hospital (min)	63 / 59 (29)	65 / 60 (29)	64 / 60 (28)

*All data in mean/median (SD) or percentages. Subgroup with simultaneous admissions includes consecutive trauma admissions within 10 min to the same facility*

### Level of care

Relative admission rates to level-one trauma centers were higher in multiple seriously injured trauma victims (68.2%), compared to regular conditions (57.9%, *p* < 0.001). Additionally, under these circumstances, patients were less frequently admitted to level-two trauma centers (32.4% under regular scenarios and 25.6% of patients under MULTiple conditions). However, this phenomenon was not observed in the case of SIMultaneously admitted patients. Admission to level-three trauma centers is not altered during any specific scenario (Table 3).

**Table 3:** Allocation, patient characteristics and resuscitation room diagnostics

	Individual admissions	Multiple admissions	Simultaneous admissions
Level of care (Trauma centre)			
Level one / supra-regional	57.9%	68.2%	58.4%
Level two / regional	32.4%	25.6%	32.1%
Level three / local	9.6%	9.5%	9.5%
Admission parameters			
Systolic blood pressure (mmHg)	128 (31)	128 (31)	130 (31)
Heart rate (BPM) *	89 (22)	89 (22)	90 (21)
Catecholamines *	20.3%	17.9%	16.7%
Resuscitation volume (mL) *	1487 (1750)	1326 (1560)	1366 (1642)
Serum Hemoglobin level (g/dL)	12.8 (2.5)	12.8 (2.5)	12.9 (2.4)
Temperature (^o^ Celsius) *	36.1 (1.2)	36.3 (1.0)	36.3 (1.1)
Base Excess	− 2.2 (4.7)	− 1.9 (4.6)	− 1.7 (4.1)
Platelet counts × 10^9^/L *	216 (81)	217 (76)	218 (78)
Quick´s value (%)	85 (22)	85 (22)	87 (21)
Partial prothrombin time (seconds) *	32 (17)	31 (17)	31 (19)
Imaging			
CT-scanning	88.2%	89.3%	88.2%
Sonography of the abdomen	83.6%	82.4%	83.8%
Chest x-ray	44.0%	39.7%	40.6%
Times to Imaging			
*Chest X-ray (min)*	14.5/8	15.4/9	16.8/10
*Sonography (min)*	5.9/4	6.4/5	7.4/5
*First CT (min)*	24.0/20	25.9/20	28.3/22
Total time in the ER* (min)	64.7/20	65.9/52	68.2/55
*Applied initial surgical concept*			
Damage control orthopedics *	8.6%	9.0%	8.1%

*All time data with mean / median (standard deviation); CI* = *confidence interval, BPM* = *beats per minute*

^*^available only for patients documented with the standard dataset

### Resuscitation room characteristics, diagnostics and procedures

Admission hemodynamics and metabolic status did not differ between groups. CT-scanning was performed in nearly 90% of cases and in all conditions. However, the time from admission to imaging was increased by 2–4 min in the case of MULTiple or SIMultaneous admissions, compared with individual admissions.

When comparing the duration of other imaging, a similar trend was observed for chest x-ray and sonography of the abdomen. Chest X-ray imaging seemed to be applied less frequent (40% vs. 44%, Table 3) in the case of MULTiple admissions. The average time in the resuscitation room was slightly increased in the case of MULTiple and SIMultaneous admissions (65.9 and 68.2 min vs. 64.7 min in regular admissions). The utilization of damage control strategies and discharge pattern from the ED was similar in all three subgroups considered.

### Outcome

No relevant differences in transfusion requirements were observed between the groups. Regarding mortality the best outcome is seen in patients treated SIMultaneously. However, after adjustment for relevant risk factors summarized according to RISC II score, observed outcome was nearly identical with the respective prognosis (Table 4). In MULTiple admitted patients, the outcome was favorable for both the first patient (*n* = 2107; observed 12.0%; expected 11.8%) and for second patient who was admitted during the ER treatment of the first (*n* = 2095; observed: 10.8%; expected: 11.3%).

**Table 4:** Outcome

	Individual admissions	Multiple admissions	Simultaneous admissions
Destination after ER*			
Emergency intervention	4.2%	4.8%	4.7%
Operation room	36.8%	35.2%	36.7%
Intensive care	50.9%	51.3%	49.2%
Ward	8.0%	8.7%	9.4%
*Blood transfusion*			
(units of pRBC until ICU)			
None	87.4%	87.8%	87.9%
1–9	10.1%	9.6%	9.6%
≥ 10	2.5%	2.5%	2.5%
Mortality in hospital	12.1%	10.8%	9.6%
24-h mortality	6.2%	5.6%	5.1%
Observed / expected mortality**			
No. of cases	117,754	4,202	1,526
Observed mortality	13.1%	11.4%	10.6%
95% CI for mortality	[12.9—13.3]	[10.4—12.4]	[9.1—12.2]
RISC II prognosis	12.8%	11.5%	10.2%

*All time data with mean / median (standard deviation); CI* = *confidence interval, BPM* = *beats per minute*

^*^available only for patients documented with the standard dataset

^**^primary admitted cases with RISC II only; no early transfer out

## Discussion

The current study demonstrates that.

1. Simultaneous treatment of seriously injured patients in German institutions is rare; it occurs in only 3.4% of trauma admissions within the TR-DGU.
2. Patients admitted under MULTiple admission scenarios have more often been involved in traffic accidents, have craniocerebral injuries and an impaired neurological status less often, and are more frequently transferred to a level-one trauma center than with regular admissions.
3. Diagnostics in multiple admission scenario situations is slightly altered and takes longer. Nevertheless, management and outcome in patients admitted in parallel to other trauma victims is similar to individual trauma admissions.

In the USA, 9% of trauma cases are considered as MCI-situations, an even more profound category of simultaneous trauma admission conditions [14]. In the current study, about 3.4% of trauma patients were admitted under multiple admission conditions. This difference can be explained by (1) variation in distribution of trauma facilities in metropolitan areas between both regions [10, 24, 25], (2) differences in definitions of MCIs [24, 25] or, (3) striking differences in occurrence of fire-arm incidents (< 5% of cases in the current study are due to penetrating trauma, whereas over 60 percent of MCI-patients in the US-study from Shoher et al. were suffering from penetrating trauma [14]).

Despite comparable injury severity, injury patterns differ slightly between groups and individually admitted trauma patients were more often diagnosed with craniocerebral injuries. This also explains the higher pre-hospital intubation rates and larger number of patients with an altered GCS score. Conversely, patients admitted simultaneous with other trauma victims had more thoracic injuries diagnosed. As anticipated, pre-hospital care was, except for different intubation rates, comparable between all groups as trauma patients are generally being treated by separate trauma teams.

Our data further show that patient allocation is altered in the case of parallel trauma cases. Under MULTiple admission scenarios, patients are more likely to be admitted to a level-one trauma center than under normal conditions. This is at the expense of level-two trauma centers which receive comparatively less patients in these cases. As level-one trauma centers are required to be able to manage the parallel treatment of multiple severely injured patients, it may be practical in some situations (i.e., car accidents) to cluster patients accordingly and admit them to the same institute. Interestingly, this discrepancy was not observed in those situations in which patients were admitted simultaneously (within 10 min). Level-three trauma center admission rates are less affected, as their role in the treatment of severely injured patients in our region is minimal.

In scenarios of multiple severely injured trauma victims, adequate patient distribution is vital. Adequate distribution entails patient allocation based on optimal matching of individual patient demands with available institutional capacities in a specific region. Trauma systems are the key factors in ensuring adequate patient distribution. The German national trauma network aims to ensure trauma coverage for the entire geographic area of Germany and to achieve optimal regional and national allocation of trauma facilities. By defining strict triage criteria for hospitals with different trauma care levels, patient distribution can be optimized and mismatches in trauma care supply and demand can be minimized [26]. This is essential, both in singular and multiple trauma situations. In both scenarios, well-designed institutional protocols are mandated and, in our view, these protocols should be practiced on a regular basis through simulation training of involved personnel [27]. This recommendation is based on studies showing that programs regularly utilizing inter-professional simulation of pre- and in-hospital trauma care are associated with better overall outcomes in severe trauma. Furthermore, in order to improve quality of care, it is essential to analyze cases of multiple trauma admissions retrospectively by debriefing sessions and/or scientific reporting [27–29].

Regarding imaging, we demonstrated that X-ray imaging was performed less frequently on patients from the MULTiple admission group when compared with regular admissions. This can be explained by the larger percentage of patients with thoracic trauma and the subsequent need for CT-scanning. CT-scanning habits in our investigation did not differ between groups, which is in contrast to a study from Aylwin et al. in which they described minimal utilization of computed tomography imaging during the casualty-receiving phase of a MCI. They further observed a trend toward more liberal use of ultrasound investigations in trauma victims during MCI-conditions [24], whereas our study did not demonstrate altered utilization of ultrasound diagnostics.

Processing times in our study, however, were affected by the admission of multiple trauma patients. Times until CT-scanning was performed, increased in the case of multiple trauma admissions (and increased even more prominently in SIMultaneously admitted patients). Prolonged waiting times for imaging, most likely also affected the total time that patients spend on the emergency department. The encountered increased processing times are therefore most likely due to the limited availability of CT-scanning devices and the need to transfer patients from the table to the bed, as well as cleaning and preparing the CT-scanner in between investigations.

Several logistical improvements have recently been shown to improve the efficiency of the CT-scanning procedure in trauma. First, Huber-Wagner et al. showed that a short distance between the CT scanner and the emergency department (defined as less than 50 m) is associated with improved outcome in severely injured patients [30]. Similar findings by Saltzherr et al. showed that a CT scanner located in the emergency department as opposed to the radiology department was associated with improved workflows [31]. Further, hybrid shock rooms equipped with an angio-embolization suite (e.g., active bleeding in pelvic injuries) can further improve efficiency and outcome in severe trauma [32, 33]. However, not all trauma centers are able to fully implement these optimized features in their emergency departments. In order to overcome this discrepancy, especially since high-volume centers are associated with better polytrauma outcomes than smaller volume centers, adequate pre-hospital triage and well-designed trauma networks are essential [34, 35].

Of note, the impact of the encountered 10% time difference between regular and simultaneous trauma admissions on outcome was not determined by the current study, and therefore, the clinical relevance of this finding remains unclear and allows for speculation. Future studies and validation in other datasets are indicated.

The current study was the first to show that simultaneous admission of seriously injured trauma cases does not alter the utilization of treatment concepts. All relevant factors involved in decision making adapted from safe definitive surgery (SDS) recommendations were comparable between groups [36]. In line with SDS-principles, application and timing of definitive care for orthopedic fractures also did not differ between the study groups.

These findings, however, are in contrast with a study from Gonzalez et al. [37] in which during an MCI in France, an evident trend toward increased utilization of damage control orthopedics was observed.

Interestingly, mortality rates are comparable between groups and no relevant differences were found in RISC-analyzes. These findings are in line with a study from Shoher et al. in which trauma patients admitted simultaneously with other patients to their urban level-one trauma center had comparable outcomes with individual admissions [14]. Ball et al. further demonstrated that ICU-stay, morbidity and mortality were not impaired in MCI-patients admitted to a single regional trauma center. Similar to our findings, they also found that processing times were increased in MCI cases compared to regular trauma admissions [13].

Our study has some limitations. As the TraumaRegister DGU® includes large numbers of patients, their validity is limited due to the identification of statistical differences without clinical relevance. Therefore, statistic testing was only performed for specific analysis with limited datasets.

In conclusion, the current study underlines the rarity of the simultaneous admission of seriously injured trauma patients in Germany. This multicenter study further reveals specific injury patterns, the distribution of patients over different types of trauma centers in multiple trauma patient scenarios, compared with regular admissions. Moreover, simultaneous admission of multiple patients is associated with decreased efficiency of diagnostics but does not worsen outcome. Upcoming research should focus on interventions aimed to further optimize preparedness for multiple admission scenarios and thereby improving outcome under these specific conditions.
